# Aortic penetration due to a fish bone: a case report

**DOI:** 10.1186/s13019-020-01325-6

**Published:** 2020-10-02

**Authors:** Daming Jiang, Yi Lu, Yigong Zhang, Zhanglong Hu, Haifeng Cheng

**Affiliations:** 1 Department of Cardiovascular Surgery, the Second Affiliated Hospital, Zhejiang University School of Medicine, 88 JieFang Road, Hangzhou, P.R. China 310009; 2 Department of Cardiology, the Second Affiliated Hospital, Zhejiang University School of Medicine, Hangzhou, China

**Keywords:** Aortoesophageal fistula, Esophageal foreign body, Esophageal perforation, Aortic penetration, Mediastinitis, Aortic infection, Thoracic endovascular aortic repair, Surgery, Case report

## Abstract

**Background:**

Aortoesophageal fistula (AEF) caused by an esophageal foreign body is a life-threatening crisis, with rapid progress and high mortality. The first case of AEF was reported in 1818, but the first successfully managed case was not until 1980. Although there have been some reports on this condition, in most cases, the aorta was invaded and corroded due to its adjacent relationship with the esophagus and subsequent mediastinitis. To date, few reports have described an aortic wall directly penetrated by a sharp foreign body, likely because this type of injury is extremely rare and most patients cannot receive timely treatment. Here, we present a rare case of a fish bone that directly pierced the aorta via the esophagus.

**Case presentation:**

A 31-year-old female experienced poststernum swallowing pain after eating a meal of fish. Gastroscope showed a fishbone-like foreign body had penetrated the esophagus wall. Computed tomography revealed that the foreign body had directly pierced the aorta to form an AEF. Surgery was successfully performed to repair the aorta and esophagus. The postoperation and follow-up was uneventful.

**Conclusions:**

For the treatment of foreign bodies in the esophagus, we should be alert of the possibility of AEFs. The effective management of AEFs requires early diagnosis and intervention, as well as long-term treatment and follow-up, which still has a long way to go.

## Background

Aortoesophageal fistula (AEF) caused by an esophageal foreign body is a life-threatening crisis, with rapid progress and high mortality [1]. Although there have been some reports on this condition, in most cases, the aorta was invaded and corroded due to its adjacent relationship with the esophagus and subsequent mediastinitis. Here, we present a rare case of a fish bone that directly pierced the aorta via the esophagus.

## Case presentation

**Fig. 1:**
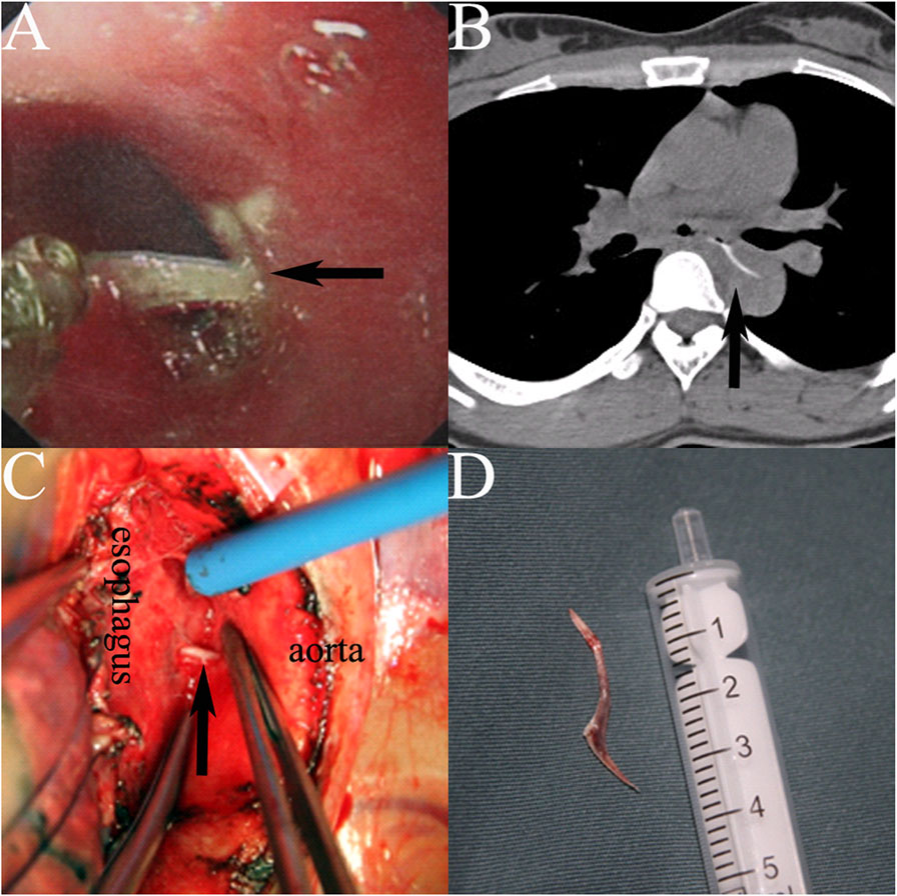
**a** A typical picture of gastroscopy demonstrats the fish bone (arrow) piercing into the esophagus wall; **b** A CT shows a fish bone (arrow) in esophagus piercing directly into the thoracic aorta. **c** Fish bone (arrow) piercing from esophagus into the aorta (Intraoperative vision). **d** Fish bone after removal

A 31-year-old female experienced poststernum swallowing pain for 2 days after eating a meal of fish. She denied any other symptoms such as fever or hematemesis. Gastroscope demonstrated a fishbone-like foreign body penetrating the esophagus wall 30 cm from the incisor (Fig. 1a). A further chest computed tomography (CT) scan revealed a sharp foreign body with a bone-like CT value just below the tracheal bifurcation, which had penetrated the thoracic aorta, resulting in mild pneumomediastinum and exudative changes around the esophagus (Fig. 1b). She was diagnosed with an AEF and referred for emergent open surgery.

**Fig. 2:**
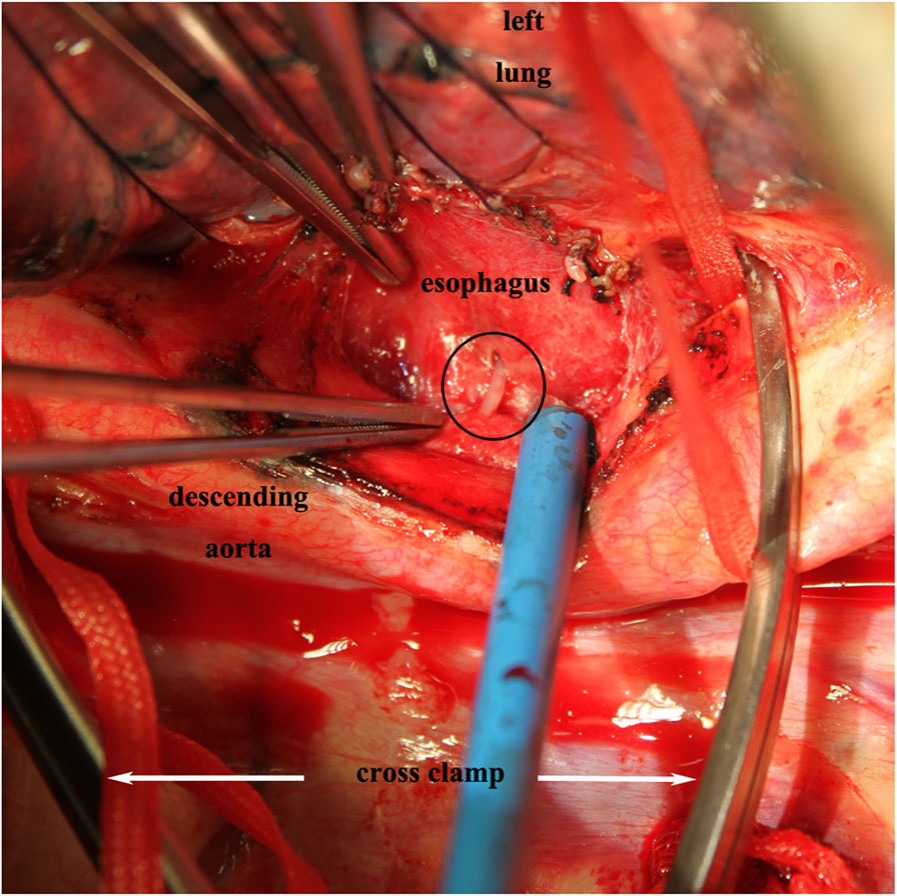
Intraoperative picture shows the operative procedure (the circle presenting the fish bone)

The patient was placed in a right lateral position. A double-lumen tracheal tube was used to facilitate ventilation of the right lung during the operation. Extracorporeal circulation was established via femoral arterial and venous cannulas. A left posterolateral thoracotomy through the fifth intercostal space was performed to gain access to the esophagus and descending aorta. The upper and lower normal aorta was isolated from the lesion area and blocking bands were set. Then, the mediastinal pleura was opened and the tissue adjacent to the lesion was separated. A long sharp fish bone emerged, which had penetrated the left esophageal wall approximately 2 cm below the tracheal carina level and pierced the descending aorta (Fig. 1c). The surrounding tissues had good vitality with mild edema. No obvious abscess was noted. After clamping the proximal and distal aorta, the contaminated tissue around the fish bone was removed and sent for culture (Fig. 2). After removal of the fish bone (Fig. 1d) and thorough debridement and irrigation with diluted polyvinylpyrrolidone-iodine and physiological saline solution, the injured aorta was repaired with 4–0 prolene suture, and the perforated esophagus was repaired with 3–0 absorbable coated Vicryl Plus antibacterial suture with the surrounding tissue flap. After repeated flushing, a drainage tube was placed in the mediastinum, and the chest was closed after the placement of another drainage tube in the chest cavity. Then, the patient was changed to the supine position and underwent laparoscopic jejunostomy. The patient recovered smoothly after the operation without complications and has lived freely for 5 years.

## Discussion and conclusions

AEF is a rare and dangerous condition, with a mortality rate between 40 and 60% [1, 2]. The first case of an AEF was reported in 1818, but the first successfully managed case was not until 1980 [3]. The common causes of AEFs include aortic aneurysms, esophageal foreign bodies and malignancy. To date, few reports have described an aortic wall directly penetrated by a sharp foreign body, likely because this type of injury is extremely rare and most patients cannot receive timely treatment.

AEF caused by an esophageal foreign body is often concealed. Patients may present with severe mediastinitis, eroding the adjacent aorta to form a pseudoaneurysm. Most patients have a clear history of an esophageal foreign body, accompanied by symptoms such as dysphagia, fever, and even Chiari triad (poststernum pain, sentinel hematemesis, and massive bleeding after an asymptomatic intermittent period). It is necessary to strive to transfer these patients to an experienced center within the golden therapeutic window (the period from sentinel hematemesis to massive bleeding, which may last several hours to months).

Once AEF is diagnosed, emergent surgery is needed [1, 4]. There is currently no unified ideal treatment for AEF caused by an esophageal foreign body. The goals of surgery include controlling the bleeding, repairing the aorta, removing mediastinal infection, repairing the esophageal defect and reconstructing the digestive tract.

Whether thoracic endovascular aortic repair (TEVAR) can be used as a definitive treatment for AEFs is very controversial [1, 5]. Many successful TEVAR cases have required additional thoracic surgeries, such as thoracoscopic or open surgery for mediastinal debridement and drainage, and esophageal repair [4, 6]. Most of the available evidence has shown that TEVAR is the first choice for rescuing patients with unstable hemodynamic parameters [1, 5]. Subsequent open surgery is recommended within 1 week after TEVAR to avoid infection of the implanted stent.

Compared with TEVAR, aortic repair with open surgery is more effective [1]. According to the size of the aortic defect, patch repair or artificial graft replacement is selected. Direct suture is suitable for small aortic perforations with no obvious signs of infection. It is recommended that a left thoracotomy be performed using extracorporeal circulation, such as femoral arterial and venous bypass, left heart bypass, etc. If the aortic involvement is wide and the arch branches are severely affected, deep hypothermia arrest may be needed [7]. After exposure of the aorta, the range and extent of the involving aorta should be carefully evaluated. The infected aorta around the fistula should be debrided and removed to eliminate potential reinfection. Compared with artificial graft, homograft has certain advantages in infectious aortic replacement [5, 7].

Esophageal perforation caused by a foreign body generally requires surgical repair [8]. There is currently no consensus on direct esophageal repair or partial resection. The surgical plan depends on the location of the perforation, the characteristics of the foreign body, and the severity of the mediastinitis [8]. Direct repair is suitable for patients with early detection of esophageal perforation, esophageal infection with few necrotic tissues, and limited mediastinal inflammation [8]. Postoperative swallowing function is better with direct repair than with resection. However, most esophageal perforations are not detected until the mediastinitis is obvious. As a result, more surgeons choose to perform esophagectomy to reduce the risk of reinfection; they also tend to reconstruct the digestive tract in stages and make the anastomosis on the neck.

The pedicled omentum can be used to wrap the repaired aorta or esophagus and fill the cavity after debridement. It has a great advantage in the treatment of infectious diseases such as AEFs, because it can isolate the infection and significantly reduce reinfection and mortality [5, 9, 10]. The pedicled intercostal muscle flap [11] and sternocleidomastoid muscle flap [12] have similar effects.

Although the clinical management of AEFs has made great progress in the past few decades, the postoperative mortality is still very high. We consider that the AEFs caused by an esophageal foreign body rarely directly penetrate the aorta and often has mild mediastinitis; thus, we directly repaired the aorta and esophagus without pedicled omentum flap wrapping. In addition, patients with AEFs generally need to fast for a long period of time, and jejunostomy has obvious advantages, with a lower incidence of long-term complications; this procedure is now used routinely at our center. Our therapeutic strategy resulted in satisfactory short- and long-term outcomes.

In conclusion, for the treatment of foreign bodies in the esophagus, we should be alerted to the possibility of AEFs. The effective management of AEFs requires early diagnosis and early intervention, as well as long-term treatment and follow-up, which still has a long way to go.

## Data Availability

The datasets of the current study are available from the corresponding author upon reasonable request.

## Abbreviations

AEF: Aortoesophageal fistula
CT: Computed tomography
TEVAR: Thoracic endovascular aortic repair
